# *Mycobacterium avium* subspecies *hominissuis* in Crohn’s disease: a case report

**DOI:** 10.1093/gastro/gov054

**Published:** 2015-10-27

**Authors:** Peilin Zhang, Lawrence M Minardi, J Todd Kuenstner, Rusty Kruzelock

**Affiliations:** 1 PZM Diagnostics, LLC., Charleston, WV, USA; 2 Department of Pathology, Charleston Area Medical Center, Charleston, WV, USA; 3 West Virginia Regional Technology Park, Charleston, WV, USA

**Keywords:** *mycobacterium paratuberculosis*, Crohn’s disease, infective colitis

## Abstract

We have cultured *Mycobacteria avium* subspecies *hominissuis* (MAH) from the blood of a patient with Crohn’s disease. The patient is a 21 year-old-female with a diagnosis of Crohn’s disease for two years. She had been treated with corticosteroids and Humira for six months. A blood specimen was cultured in a specialized medium, and there was visible bacterial growth present in the liquid culture medium after eight weeks. PCR analysis of the bacterial growth and subsequent direct sequencing of the PCR amplicon confirmed the presence of MAH. The significance of this finding is discussed.

## Introduction

Crohn’s disease is a chronic inflammatory disorder involving the gastrointestinal tract. Crohn’s disease is currently considered an idiopathic autoimmune condition, and immune suppressants are generally used for the treatment of the disease [1]. In the past 60 years, many investigators have sought a pathogen causing the disease, and various bacteria and viruses have been isolated and reported [2,4]. For many years, *Mycobacterium avium* subspecies *paratuberculosis* (MAP), a known pathogen in Johne’s disease (a chronic wasting condition in cattle and sheep) [5] has been suspected to cause Crohn’s disease [6]. The role of MAP in Crohn’s disease is controversial [6,7], but two meta-analyses have concluded that a majority of patients with Crohn’s disease have evidence of MAP infection [8,9].

MAP is a notoriously slow-growing pathogen under routine cultural conditions [10,11]. Therefore, we sought to develop a more rapid culture method for MAP and/or other bacteria from the blood of Crohn’s patients. As a result of this effort, we have cultured *Mycobacterium avium* subspecies *hominissuis* (MAH) from the blood of a Crohn’s patient. To our knowledge, we are the first to report MAH being isolated from a Crohn’s patient. The significance of this finding is discussed.

### Case report

**Figure 1.**
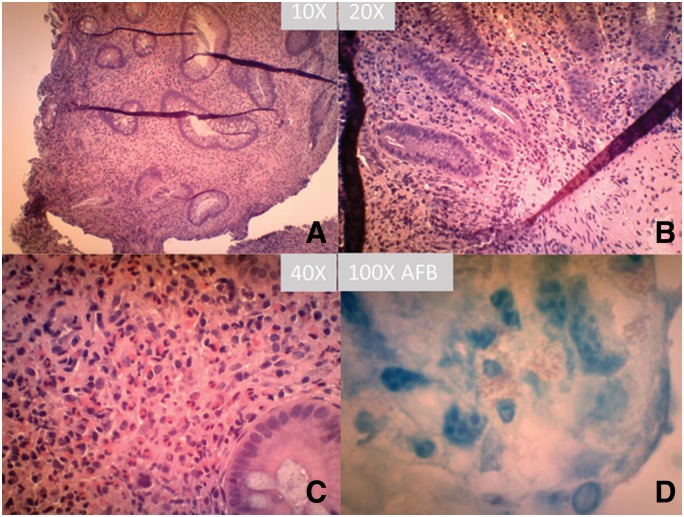
Hematoxylin and eosin (H&E) stains of the colonic biopsy at 10× (**A**), 20× (**B**) and 40× (**C**) magnification with classic features of Crohn’s disease. (D) Acid-fast (AFB) stain of the biopsy tissue.

The patient is a 21-year-old local female from the Midwest region of United States with a known history of Crohn’s disease for two years. The patient’s symptoms had been difficult to control in the past, and most recently she had been on a corticosteroid (prednisone 40 mg daily) in addition to a monthly Humira injection over the past six months. Over the past year, the patient continued to have severe colicky cramping abdominal pain, nausea and intermittent diarrhea. She was recently evaluated for worsening abdominal pain and cramping, and colectomy was considered because of her poor response to therapy. A recent colonoscopy was performed by the gastroenterologist, and active disease was seen in the cecum, rectum and sigmoid colon. The patient was also found to have eosinophilia with 15% eosinophils in her blood differential count. Because of the eosinophilia and history of drinking well water, a search for parasites such as *Strongyloides* was performed, and there was no parasite within the stool. The left and right colon biopsies showed features of active colitis including cryptitis, crypt abscesses and increased lymphocytic and eosinophilic infiltration in the lamina propria (Figure 1A-C). Small, loosely formed granulomas were identified. GMS silver stain and acid-fast stain were performed, and there were apparent small oval/rod acid-fast bacteria within the histiocytes and the lamina propria (Figure 1D). The histopathological features were consistent with inflammatory bowel disease (Crohn’s disease). There was no morphologic evidence of eosinophilic colitis.

**Figure 2.**
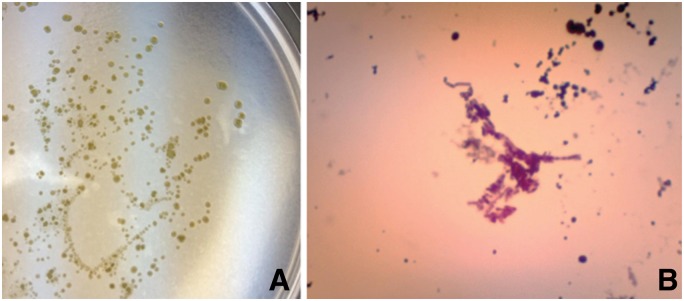
Subculture of *Mycobacteria avium* subspecies *hominissuis* (MAH) from the blood (**A**) and its acid-fast staining (100×, **B**)

A blood culture was sent to us and performed in specialized medium based on Middle brook 7H9 with supplements of oleic acid, albumin, dextrose and catalase, from BD Biosciences (OADC) and mycobactin J [12]. Briefly, the medium was prepared in our own laboratory, and blood culture was performed using the following method developed in our laboratory. One 4-ml purple-top tube of blood was centrifuged at 5000 g for 10 minutes in a 15-ml centrifuge tube to separate the plasma and the cellular components. The plasma was removed from the centrifuge tube and stored at −20°C in a freezer for MAP antibody titer testing for the presence of MAP using the whole cell extract of MAP. A sterile red blood cell lysis buffer (ammonium chloride buffer containing 0.8% ammonium chloride, 0.08% sodium bicarbonate, 0.037% disodium EDTA) was added to the centrifuge tube in 4× volume (10 ml), and the cellular blood components were resuspended in the red cell lysis buffer by turning the capped tube upside down multiple times until no visible clumps were present. The nucleated cells were collected by centrifugation at 6000 g for 10 minutes, and the lysed red cells and supernatants were emptied into a biohazard container. The nucleated cell pellet was resuspended in a 4 ml liquid medium of Middlebrook 7H9 supplemented with 10% OADC and mycobactin J at 2 µl/ml. Two aliquots of the resuspended nucleated cells in liquid medium were removed with sterile pipette and planted on two separate solid media plates, one for induction of the cell-wall deficient form of MAP (spheroplasts) and one for maintenance culture. The solid induction medium contained essentially identical components with the solid maintenance medium and liquid medium except for addition of 20% sucrose and 3% glycerol. After the blood culture was incubated for two weeks at 37°C, an aliquot of culture was removed, and an acid-fast stain was performed (Figure 2). The cellular components of culture, including the nucleated cells from the blood and anything growing during the two-week culture period, were collected by centrifugation at 12 000 g for five minutes. The cultured material was washed with PBS buffer (pH 7.4) once and resuspended with 0.5 ml 100% acetone. The cultured material was collected by centrifugation at 12 000 g for five minutes and resuspended in 500 µl pH 7.6 TE buffer (10 mmol/L Tris, 1 mmol/L EDTA, pH 7.6) and 0.5% SDS. Proteinase K (five units) were added and incubated at 65°C overnight. The genomic DNA was isolated by phenol/chloroform/isoamyl-alcohol extraction and alcohol precipitation as described elsewhere [13]. The genomic DNA was used for PCR analysis using primers specific for IS900, IS901, IS1245 and 16s rDNA (Table 1) [6,14–16]. The PCR amplification was performed in 50 μl volume using 94°C for 30 seconds, 55°C for 30 seconds and 72°C for 45 seconds for 40 cycles using the PCR Core-kit (Sigma Aldrich). The PCR amplicon was visualized on 1.2% agarose gel electrophoresis. The PCR amplicons were subjected to DNA sequencing analysis to Eurofins Genomics sequencing services, and the amplified DNA sequences were submitted for BLAST analysis against Genbank nucleotide sequences at NCBI (http://blast.ncbi.nlm.nih.gov/).

**Table 1.** PCR primer sequences

Primers	DNA sequences (5’–3’)
IS901-F	GGA TTG CTA ACC ACG TGG TG
IS901-R	GCG AGT TGC TTG ATG AGC G
IS1245-F	GAG TTG ACC GCG TTC ATC G
IS1245-R	CGT CGA GGA AGA CAT ACG G
16S-F	GAG GAA GGT GGG GAT GAC G
16S-R	AGG CCC GGG AAT GTA TTC AC
IS900-F	CTT TCT TGA AGG GTG TTC GG
IS900-R	GAG GTC GAT CGC CCA CGT GA

**Figure 3.**
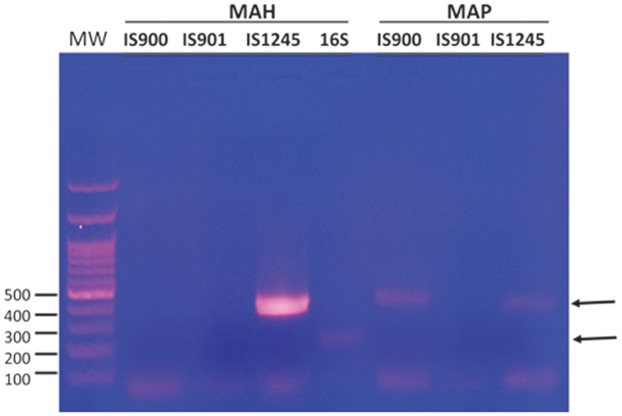
PCR amplicons using various primers specific for IS900, IS901 and IS1245 for *Mycobacterium avium* complex. 16S depicts the primers for 16S rDNA. *Mycobacterium avium* subspecies *paratuberculosis* (MAP) strain was from ATCC (cat: 43545).

PCR primers for 16S rDNA and IS1245 produced distinct bands for the culture, and the PCR amplicons were sent for direct DNA sequencing. The amplicon sequence using 16s rDNA primers matched both MAP (100%, GenBank: CP010114.1) and MAH (100% GenBank AP012555.1), and the amplicon sequence using IS1245 primers also matched both MAP and MAH (99%, GenBank AP012555.1). However, PCR analysis using the primer set derived from IS900 was negative, indicating that the isolate was not MAP. Further testing with PCR primer sets for IS901 and IS1245 showed that only IS1245 was positive, confirming the identity of MAH (Figure 3).

## Discussion

In this case, using the culture method described above, we cultured MAH from the blood of this Crohn’s patient but did not recover MAP. MAH is one of the closest members of *Mycobacterium avium* complex to MAP, in additional to *Mycobacteria avium* subspecies *avium* (MAA) and *silvaticum* (MAS) [17]. MAH is present in the environment in soil and water and is a known pathogen in animals such as pigs, dogs, deers and horses [17–19]. There are reports of MAH isolates from the lymph nodes of humans with lymphadenitis [20]. To our knowledge, there is no report to date of MAH from Crohn’s patients. There is a report of MAH isolated from the gastrointestinal tract of deer [21].

The presence of MAH in a Crohn’s patient raises questions of the role of MAH in Crohn’s disease. It is possible that MAH is a secondary infectious agent in patients with immune suppressants (i.e. an opportunistic pathogen). It is equally possible that MAH is the pathogenic agent in this patient with Crohn’s disease. In fact, MAH is a more common isolate from cancer patients or HIV-positive patients, but the clinical manifestation of MAH in cancer patients or HIV-positive patients is mostly respiratory/pulmonary [22]. Regardless of the role of MAH in the pathogenesis of Crohn’s patients, the presence of MAH in the blood of these patients indicates a mycobacteremia, and this condition requires antibiotic treatment for the affected patients.

Genetically there are multiple gene allelic mutations associated with Crohn’s disease, and there is a significant overlap of genetic susceptibility loci of Crohn’s disease and mycobacterial infections [23]. These genetic loci susceptible to mycobacterial infections include *Mycobacterium tuberculosis* and *Mycobacterium leprae.* Other mycobacterial infections are possible due to the host genetic changes of Crohn’s disease. We suspect that MAH is one of the causative agents of Crohn’s disease.

## References

[1] Lichtenstein GR, Hanauer SB, Sandborn WJ Management of Crohn’s disease in adults. Am J Gastroenterol 2009;104:465–83; quiz 464,484. 19174807 10.1038/ajg.2008.168

[2] Aronson M. D., Phillips C. A., Beeken W. L., Forsyth B. R. Isolation and characterization of a viral agent from intestinal tissue of patients with Crohn's disease and other intestinal disorders. Prog Med Virol 1975;21: 165–76. 1208878

[3] Parent K., Mitchell P. Pseudomonas-like group Va bacteria in Crohn's disease. Gastroenterology 1978;75: 765. 710845

[4] Graham D. Y., Markesich D. C., Yoshimura H. H. Mycobacteria and inflammatory bowel disease. Results of culture. Gastroenterology 1987;92: 436–42. 3792780 10.1016/0016-5085(87)90139-9

[5] Behr M, Collins DM Paratuberculosis: Organism, Disease, Control. Wallingford, UK: CAB Int, 2010.

[6] Naser SA, Ghobrial G, Romero C Culture of *Mycobacterium avium* subspecies *paratuberculosis* from the blood of patients with Crohn’s disease. Lancet 2004;364:1039–44. 15380962 10.1016/S0140-6736(04)17058-X

[7] Parrish NM, Radcliff RP, Brey BJ Absence of *mycobacterium avium* subsp. *paratuberculosis* in Crohn’s patients. Inflamm Bowel Dis 2009;15:558–65. 19058231 10.1002/ibd.20799

[8] Feller M., Huwiler K., Stephan R., Altpeter E., Shang A., Furrer H., Pfyffer G. E., Jemmi T., Baumgartner A., Egger M. Mycobacterium avium subspecies paratuberculosis and Crohn's disease: a systematic review and meta-analysis. Lancet Infect Dis 2007;7: 607–13. 17714674 10.1016/S1473-3099(07)70211-6

[9] Abubakar I., Myhill D., Aliyu S. H., Hunter P. R. Detection of Mycobacterium avium subspecies paratuberculosis from patients with Crohn's disease using nucleic acid-based techniques: a systematic review and meta-analysis. Inflamm Bowel Dis 2008;14: 401–10. 17886288 10.1002/ibd.20276

[10] Chiodini R. J., Van Kruiningen H. J., Merkal R. S., Thayer W. R. Jr., Coutu J. A. Characteristics of an unclassified Mycobacterium species isolated from patients with Crohn's disease. J Clin Microbiol 1984;20: 966–71. 6511878 10.1128/jcm.20.5.966-971.1984 PMC271485

[11] Chiodini RJ, Van Kruiningen HJ Spheroplastic phase of mycobacteria isolated from patients with Crohn’s disease. J Clin Microbiol 1986;24:357–63. 3760132 10.1128/jcm.24.3.357-363.1986 PMC268913

[12] Ghodbane R., Raoult D., Drancourt M. Dramatic reduction of culture time of Mycobacterium tuberculosis. Sci Rep 2014;4: 4236. 24577292 10.1038/srep04236 PMC3937792

[13] Sambrook J, Fritsch EF, Maniatis T Molecular Cloning: A Laboratory Manual. 2nd ed New York: Cold Spring Harbor Laboratory Press, 1989.

[14] Jeyanathan M, Alexander DC, Turenne CY Evaluation of in situ methods used to detect *Mycobacterium avium* subsp. *paratuberculosis* in samples from patients with Crohn’s disease. J Clin Microbiol 2006;44:2942–50. 16891515 10.1128/JCM.00585-06 PMC1594655

[15] Lahiri A, Kneisel J, Kloster I Abundance of *Mycobacterium avium* ssp. *hominissuis* in soil and dust in Germany - implications for the infection route. Lett Appl Microbiol 2014;59:65–70. 24612016 10.1111/lam.12243

[16] Relman DA, Loutit JS, Schmidt TM The agent of bacillary angiomatosis. An approach to the identification of uncultured pathogens. N Engl J Med 1990;323:1573–80. 2233945 10.1056/nejm199012063232301 PMC13632802

[17] Iwamoto T, Nakajima C, Nishiuchi Y Genetic diversity of *Mycobacterium avium* subsp*. hominissuis* strains isolated from humans, pigs, and human living environment. Infect Genet Evol 2012;12:846–52. 21745597 10.1016/j.meegid.2011.06.018

[18] Campora L., Corazza M., Zullino C., Ebani V. V., Abramo F. Mycobacterium avium subspecies hominissuis disseminated infection in a Basset Hound dog. J Vet Diagn Invest 2011;23: 1083–87. 21908381 10.1177/1040638711418616

[19] Kriz P., Jahn P., Bezdekova B., Blahutkova M., Mrlik V., Slana I., Pavlik I. Mycobacterium avium subsp. hominissuis infection in horses. Emerg Infect Dis 2010;16: 1328–29. 20678342 10.3201/eid1608.100097 PMC3298305

[20] Kaevska M, Slana I, Kralik P “*Mycobacterium avium* subsp. *hominissuis”* in neck lymph nodes of children and their environment examined by culture and triplex quantitative real-time PCR. J Clin Microbiol 2011;49:167–72. 21084514 10.1128/JCM.00802-10 PMC3020421

[21] Glawischnig W, Steineck T, Spergser J Infections caused by *Mycobacterium avium* subspecies *avium, hominissuis*, and *paratuberculosis* in free-ranging red deer (Cervus in Austria, 2001‐2004. J Wildl Dis 2006;42:724–31. 17255438 10.7589/0090-3558-42.4.724

[22] Tran QT, Han XY Subspecies identification and significance of 257 clinical strains of *Mycobacterium avium*. J Clin Microbiol 2014;52:1201–6. 24501026 10.1128/JCM.03399-13 PMC3993508

[23] Jostins L, Ripke S, Weersma RK Host-microbe interactions have shaped the genetic architecture of inflammatory bowel disease. Nature 2012;491:119–24. 23128233 10.1038/nature11582 PMC3491803

